# Impact of social franchising on TB contact investigation and uptake of TB preventive therapy

**DOI:** 10.5588/pha.24.0054

**Published:** 2025-09-03

**Authors:** M. Sheshi, B. Odume, O. Chukwuogo, C. Ogbudebe, I. Gordon, S. Useni, N. Nwokoye, M. Bajehson, D. Nongo, R. Eneogu, A. Ihesie, U. Omo-Emmanuel, S. Wadok, R. Furth, C. Anyaike

**Affiliations:** ^1^ KNCV Tuberculosis Foundation Nigeria, Abuja; Nigeria;; ^2^ United States Agency for International Development, Abuja, Nigeria;; ^3^ University of Edinburgh UK;; ^4^ John Snow Incorporated (JSI) Boston, United States;; ^5^ National Tuberculosis and Leprosy Control Programme, Federal Ministry of Health Abuja, Nigeria.

**Keywords:** tuberculosis, TB prophylaxis, Nigeria, TPT

## Abstract

**BACKGROUND:**

TB continues to pose significant public health challenges in high-burden regions such as Kano State, Nigeria, where private health sector engagement in TB control is notably lacking. The Social Franchising for TB Contact Investigation (SOFT) model was introduced to leverage private healthcare to increase the reach and efficacy of TB control efforts.

**METHODS:**

This nine-month project supported mapping health facilities, training of community health workers and systematic TB contact screening. The SOFT model aimed to enhance TB control by integrating private healthcare facilities and community-based organisations to improve TB yield, contact investigation and uptake of TB Preventive Therapy (TPT).

**RESULTS:**

The project showed a consistent increase in TB case detection, with a significant rise in index TB cases identified and their contacts screened each quarter. There was also a marked increase in the number of household contacts screened and initiated on TPT, demonstrating the model’s effectiveness in enhancing TB control efforts.

**CONCLUSION:**

The integration of social franchising with community and private healthcare engagement presents a scalable and innovative approach to improving TB control in high-burden settings. This model contributes significantly to global TB elimination efforts by improving detection rates and TPT uptake.

TB remains a major global health challenge, causing significant morbidity and mortality worldwide.^1-4^ In Nigeria, WHO estimates the incidence rate for all forms of TB at 219 per 100,000 population, with over 98,000 TB-related deaths in 2022.^5^ Kano State, the most populous state in Nigeria, is among the regions most affected, with high incidence rates that reflect the broader public health challenges faced by the country in TB control. WHO has identified Nigeria as the 6^th^ highest TB burden globally, underscoring the urgency of enhancing TB control measures.^6^ Kano State’s struggle with TB is exacerbated by a complex interplay of factors, including socioeconomic challenges, a high population density, and limited access to quality healthcare services, particularly in rural areas.^6^ Despite its potential, the private healthcare sector in Kano State has been sub-optimally utilised in TB control efforts.^7^ This sector encompasses a wide range of healthcare providers, from formal private sector providers such as private clinics and hospitals, private standalone medical laboratories as well as pharmacies, and informal private sector providers such as patent medicine vendors, which are often the first point of contact for many individuals seeking health care.^8^ However, the engagement of these private entities in TB control has been limited by several factors, including a lack of awareness, inadequate training in TB case detection and management, and insufficient collaboration with public health initiatives.^9^ Consequently, opportunities for early TB detection and treatment in the private sector have been missed, contributing to ongoing transmission and high TB mortality rates. Furthermore, even when these private health providers are engaged in TB control, they have limited ability to carry out services such as contact investigation as they do not have the staff needed to optimally carry out such services.

Recognising these challenges, the Social Franchising for TB Contact Investigation (SOFT) project was initiated with funding from United States Agency for International Development (USAID) through John Snow Incorporated (JSI), as an innovative approach to enhance TB control in Kano State. The project aimed to leverage the state's robust private sector by implementing a sustainable social franchising model. Social franchising in healthcare applies the principles of franchising to achieve public health goals, creating networks of private healthcare providers to replicate and scale up services according to standardised quality criteria.^10^ By engaging private healthcare providers in TB detection and management, the SOFT project sought to bridge the gap in TB care, facilitating early detection, appropriate treatment, and prevention efforts. The SOFT model’s approach was grounded on the understanding that increasing the engagement of private healthcare providers in TB control could significantly enhance case finding and preventive measures. This model aimed not only to improve TB outcomes in Kano State but also to serve as a scalable and replicable strategy for TB control in other similar settings in Nigeria. Through targeted training, support, and incentives, the project aimed to build the capacity of private healthcare providers, integrating them into the broader TB control efforts and ensuring a more comprehensive and effective response to the TB epidemic in Kano State. Kano State faces a critical public health challenge with high TB incidence and mortality rates. Despite ongoing efforts, TB remains a major crisis, necessitating innovative approaches for better detection, treatment, and prevention.^11^

An assessment of the SOFT model's immediate impact on TB outcomes and its long-term sustainability and scalability potential in similar regions was carried out. The rationale for this lies in the urgent need for innovative, sustainable, and scalable TB solutions in Kano State and beyond, offering insights to shape future public health strategies. We evaluated SOFT model's impact on TB yield and prevention efforts in Kano State, Nigeria. Specifically, three key objectives. First, to determine the TB yield using the SOFT model across selected health facilities in Kano. Second, to increase the proportion of contacts of TB-positive cases screened for TB within the communities. Finally, to increase the uptake of TB preventive therapy (TPT) among eligible contacts of TB-positive cases among the target population.

## METHODS

Kano state is one of the 14 states where KNCV Nigeria implements the TB LON 1&2 project, utilising the private sector to increase TB case finding. From October 2021 to September 2022, private health facilities contributed more than half of the 16,485 TB cases diagnosed from the LON intervention in Kano state.^12^ Despite various incentives to motivate private health practitioners to investigate the contacts of index TB cases, the index patient coverage has remained below 25%. A health facility-based repeated cross-sectional design was used for the SOFT TB program. Field implementation spanned from January 2023 to September 2023 (a 9-month period), involved the line-listing of index TB cases at the health facilities, conducting TB screening of household contacts of identified TB cases, sputum or stool sample collection for presumptive TB cases and referrals for chest x-ray and escort services for newly identified TB cases and household contacts eligible for TPT for commencement on treatment.

KNCV Nigeria collaborated with the National TB Program to commence project implementation activities with routine oversight from JSI TIFA and USAID Nigeria teams. The community entry phase started with a project introductory meeting of relevant private sector associations such as the chairpersons and secretaries of the Guild of Medical and Medical Laboratory Directors, National Association of Proprietary and Patent Medicine Vendors, Association of Community Pharmacists of Nigeria, and the Association of General Private Nursing Practitioners of Nigeria. Also, the project engaged five community-based organisations (CBOs) to spearhead the contact investigation efforts based on their track record, community reach, and commitment to public health initiatives in the TB program. Each CBO played a pivotal role in mobilising and overseeing a team of 10 Community Health Workers (CHWs) to ensure coverage of the assigned LGAs and health facilities.

The project strategically mapped 668 health facilities in Kano State using the innovative hub-and-spoke model. A dedicated team of 25 enumerators employed geographical information systems (GIS) technology and a mapping checklist hosted on the SurveyCTO app for offline data capture. The mapping team were trained alongside the private sector and state TB program stakeholders to identify, categorise, and geographically map 668 health facilities across the state. Likewise, 50 CHWs, directors of each CBO and 170 private health facility DOT officers were trained to conduct effective contact investigation and implement TPT strategies. Subsequently, the CHWs engaged in line-listing of index TB cases, elicitation of household contacts from the index TB case, conducting household visits for TB screening, sample collection and movement to diagnostic facilities, referrals and linkages for newly identified TB cases and household contacts eligible for TPT in addition to documentation of all TBCI activities.

Data were methodically gathered from health facility registers, community health worker reports, and patient records. Key metrics such as the number of index TB cases found, contacts screened, TB cases diagnosed, and individuals starting TPT were rigorously documented and analysed. Descriptive statistics, which included frequencies, percentages, and trends across time, were used to summarise the data. This technique enabled a thorough knowledge of the project's impact on TB identification and prevention in the community.

The study was determined to be a non-research program evaluation. As it required no direct contact with human subjects (no interview or sample collection), and only de-identified pooled program data that formed part of the standard of care were used, informed consent was not required.

## RESULTS

**FIGURE 1.**
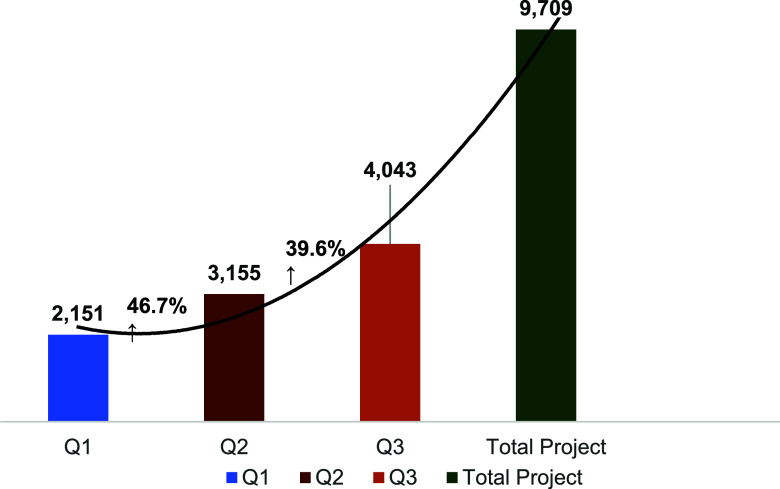
Index TB cases identified using SOFT model. Q = quarter.

**FIGURE 2.**
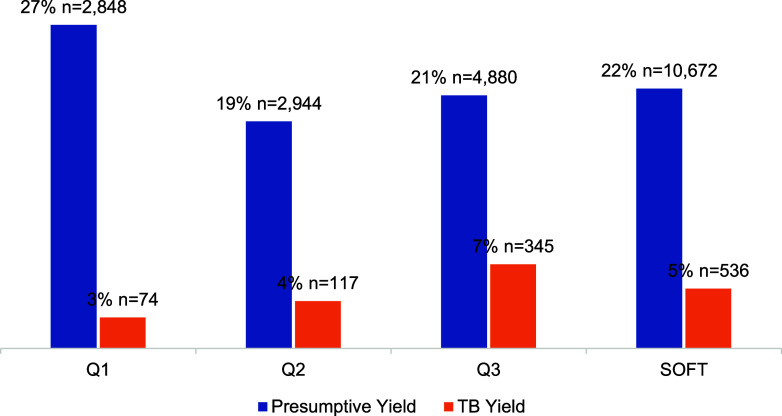
TB case detection of SOFT project, presumptive cases and TB yield. N = number; Q = quarter.

The SOFT model demonstrated a progressive increase in identifying index TB cases over the implementation period. In the first quarter (Q1), 2,151 index TB cases were identified, with a 46.7% and 39.6% increase in Q2 and Q3 (n = 3,155 and n = 4,403) respectively. Over the entire project period, 9,709 index TB cases were identified, and their contacts were subsequently screened for TB (Figure 1). After the contacts of index TB cases were screened, in Q1, 74 (3%) household contacts were diagnosed for TB, while in Q2 and Q3, 117 (4%) and 345 (7%) household contacts were newly diagnosed for TB respectively (Figure 2). Overall, only 3 people (only in Q1, 0.6%) out of all the newly diagnosed household contacts were found to have disease-resistant TB (DR-TB). These patients with DR-TB were diagnosed using the WHO-recommended rapid diagnostics (WRDs), which is a bacteriological test for rifampicin resistant TB (RR-TB) All TB cases were notified and placed on treatment using the appropriate standardized regimen (2RHZE/4RH for DSTB, and the BPaLM regimen for DR-TB), maintaining a 100% enrolment, notification and treatment initiation rates across all quarters of the SOFT project.

**FIGURE 3.**
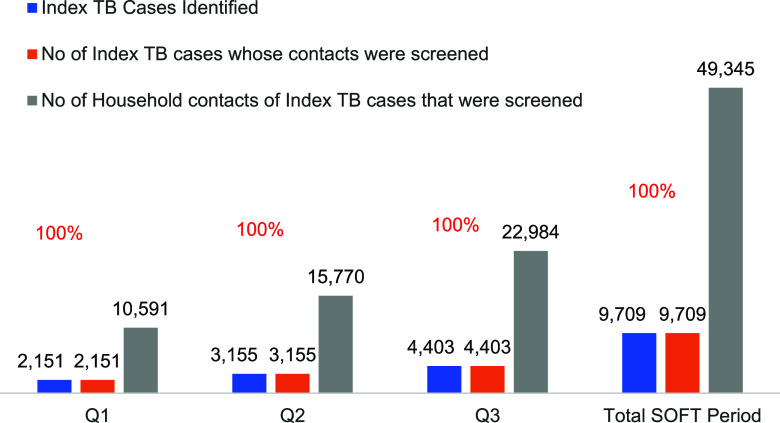
SOFT index coverage. No = number; Q = quarter.

**FIGURE 4.**
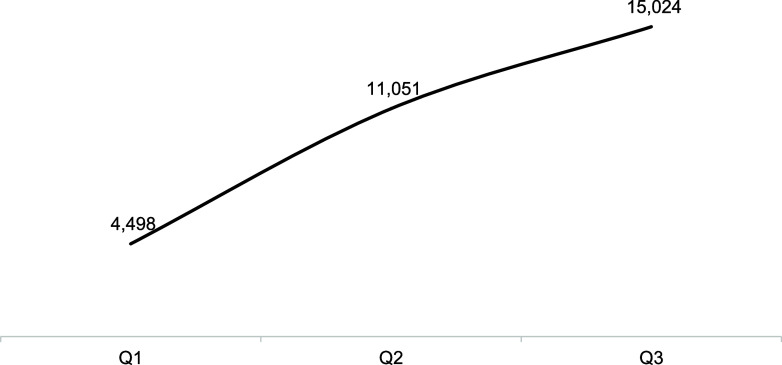
Cumulative number of household contacts placed on TB preventive therapy (TPT). Q = quarter

Analysis of the household contacts screened under the SOFT model shows a significant improvement. From 10,591 contacts screened in Q1, the number increased to 15,770 in Q2 and further to 22,984 by Q3 achieving almost 100% screening coverage (Figure 3). This consistent increase in the number of household contacts identified and screened underscored the project’s efficiency in contact tracing and the effectiveness of the strategies employed for TB control within communities. Within three-quarters of the SOFT project, TPT was significantly enhanced among eligible contacts. Cumulatively, a total of 15,024 contacts were started on TPT using the 3HR regimen (3 months of daily rifampicin and isoniazid). Starting with 4,498 contacts in Q1 and subsequently increasing to 6,553 in Q2, however, there was a slight decrease to 3,973 in Q3 (Figure 4) due to low TPT stock levels. The substantial number of contacts initiated on TPT reflect the project's success in not only identifying TB cases but also initiating preventive measures.

## DISCUSSION

The implementation of the SOFT model in Kano State, Nigeria, adopted a novel approach to TB control efforts through enhanced case finding and TPT uptake by leveraging the private healthcare sector and CBOs. The findings suggest substantial progress in improving TB case finding and increasing TPT in the selected private facilities and relevant communities. The model increased the identification of index TB cases (9,709 cases over nine months) in the selected communities before the assessment, mainly from public health facilities, a critical first step in the TB control cascade, with a consistent quarter-over-quarter increase. This success can be attributed to the project’s comprehensive approach towards engagement with private healthcare facilities and the effective use of CBOs for extensive contact tracing. It also highlights the importance of context-based strategies as the involvement of CBOs improved TPT uptake as cultural barriers within the communities were easily navigated.^13, 14^ The success of the model underscores the effectiveness of the social franchising model and the pivotal role of CBOs in extending healthcare reach, aligning with findings from similar interventions in other high-burden countries that emphasise community engagement as crucial for TB control.^15^

With regards to identification and initiation on TPT, the high number of contacts that were identified and subsequently placed on TPT suggests the effectiveness of the SOFT model through private health sector participation and a high level of community penetration that was previously unattained without the social franchising model with private healthcare providers. This is consistent with findings in the literature highlighting the critical role of TPT in TB control, particularly in settings with high transmission rates.^15^ Similarly, the SOFT model with the community at the heart of its design through CBOs and private healthcare facilities offers a viable template for TB control, resonating with global evidence that community-based approaches significantly enhance TB case finding and treatment outcomes.^16^ The model echoes the WHO's End TB Strategy, which calls for early diagnosis of TB, including active TB case finding and systematic screening of contacts and high-risk groups at the community level.^14^ The increasing number of contacts diagnosed with TB across the quarters indicates not only the model’s effectiveness in screening and evaluation but also highlights the high burden of undetected TB within the community, underscoring the importance of such targeted interventions.

Furthermore, the substantial increase in TPT uptake among eligible contacts with this model approach signifies a critical advance in TB prevention. This aligns with recent studies advocating for the expansion of TPT to reduce TB incidence and mortality, especially in high-burden settings.^17–21^ The efficiency and effectiveness of social franchising to increase TB case finding and TPT uptake as offers an innovative approach to healthcare delivery in TB control. This model, by standardising care across a network of providers in Kano, Nigeria, has shown promise in other settings. The outcomes from this project support the potential scalability of this model in other high-burden TB settings in Nigeria and other LMICs, aligning with findings from studies on social franchising for improving the quality and accessibility of healthcare services.^22–24^

The focus of this study was on impact on TB yield and prevention efforts, and it is worth noting that the study did not assess the social franchising model with regards to overall TB control (e.g. loss to follow up, or treatment success rates). Although our assessment presents promising results, there is a need for further research to explore the long-term sustainability and impact of the SOFT model on TB control and elimination. A limitation of our assessment was that it was conducted in selected health facilities in Kano State. Future studies should focus on evaluating the cost-effectiveness of social franchising in TB control, understanding the barriers and facilitators to scaling up this model, and exploring its applicability in different health system contexts.

## CONCLUSION

The SOFT model's success in improving TB case detection and TPT uptake in Kano State highlights the potential of social franchising as an innovative and effective approach to TB control. By integrating private healthcare providers and leveraging community resources, the SOFT model offers a scalable and sustainable TB prevention and treatment strategy, aligning with global health priorities and contributing valuable lessons for TB control efforts. The lessons learned from this innovative approach serve to inspire further research, policy formulation, and program implementation strategies, aiming not just to replicate its successes in Kano, Nigeria, but also to adapt and innovate upon them to meet the evolving challenges of TB control and elimination in the rest of Nigeria and other LMICs.
